# Rideshare use among parents and their children

**DOI:** 10.1186/s40621-021-00302-4

**Published:** 2021-03-01

**Authors:** Johnathon P. Ehsani, Jeffrey P. Michael, Andrea Gielen

**Affiliations:** Center for Injury Research and Policy, Department of Health Policy and Management, Johns Hopkins Bloomberg School of Public Health, 624 N. Broadway, Hampton House 555, Baltimore, MD 21205 USA

## Abstract

Motor vehicle crashes are the leading cause of death for young children. Millions of ridesharing trips are taken each day, and use of these services is predicted to increase. Therefore, it is important to examine the safety of children in these vehicles. We conducted a survey of a nationally representative sample of U.S. adults aged 18 years or older (*N* = 2017). Of the total sample, 450 respondents reported being a parent or legal guardian of children below the age of 10. Of these, 307 or 68% had ever used ridesharing. Among those who had used ridesharing, a total of 253 or 82% reported using ridesharing with their children below the age of 10 years. Among this group, rideshare use was significantly higher among individuals with college education, and in higher income households. Given that the majority of U.S. states have legislation exempting rideshare vehicles from child restraint system law coverage, our finding of high rates of rideshare use among parents suggests that a large number of children could be at risk of injury due to a lack of appropriate restraint use.

## Introduction

Prior to the COVID-19 pandemic, ridesharing companies such as Lyft and Uber provided over 4 million trips per day in urban and suburban areas (Uber Technologies Inc, 2019). In 2019, Uber released a safety report indicating that the fatal crash risk of their trips is comparable to those among adults using personal vehicles who are likely to be traveling with young children (Uber Technologies Inc, 2019; Tefft, 2017). While considerable advances have been made in child restraint use in personal vehicles (Li & Pickrell, 2018), motor vehicle crashes remain the leading cause of death for young children (Centers for Disease Control and Prevention, 2020) and the lack of restraint use has been identified as a key risk factor in fatal crashes involving children (Wolf et al., 2017). As rideshare use is predicted to increase in coming years, it is important to determine the prevalence ridesharing use among families with children.

## Methods

We conducted an online survey of a nationally representative sample of U.S. adults aged 18 years or older between December 12th and 16th, 2019. We used the general population sample (*N* = 2017) to identify our sample of interest, i.e. parents/legal guardians of children below age 10 (*N* = 450). Parents/legal guardians were asked if they have ever used rideshare (e.g., Uber, Lyft, etc.) and if yes, if their children below the age of 10 have ever been in a ride share with them. Respondents were not asked about the use of child restraints in ridesharing.

Participants were recruited from The Harris Poll opt-in panel. This is not a probability-based sample, which means an estimate of sampling error cannot be calculated. Demographic and propensity score statistical weighting were applied to adjust for respondents’ propensity to be online and to ensure the results are representative of the general population. The Johns Hopkins Bloomberg School of Public Health institutional review board deemed this study not human participants research.

## Results

From the sampling frame of 2017 U.S. adults, a total of 450 respondents reported being a parent or legal guardian of children below the age of 10. Of these, 307 or 68% had ever used ridesharing. Among those who had used ridesharing, a total of 253 or 82% reported using ridesharing with their children below the age of 10 y. Rideshare use with children was significantly higher among individuals with some college education compared to those with high school education or less (57% compared to 47%), and in households with an income of $50,000 or higher compared to those below $50,000 (60% compared to 43%). Adults between 18 and 34 years and those 35–44 years reported the highest percentage of ridesharing use (71 and 65% respectively), and these groups were also the most likely to report having children ages 9 or under (38 and 47% respectively).

## Discussion

At the time of this survey, 34 U.S. states have legislation exempting rideshare vehicles from child restraint system (CRS) law coverage (Owens, 2019). Our finding of high rates of rideshare use among parents suggests that a large number of children could be at risk of injury due to a lack of appropriate restraint use. Consistent with prior studies, rideshare use was higher among more educated and affluent segments of the population (Jiang, 2019). While we did not assess the actual use of CRS in rideshare vehicles, previous research indicates that nearly 60% of parents/caregivers reported transporting children under 5 years of age differently in rideshare than in a personal vehicle, with 37% reporting holding children on their lap and 25% letting a child ride without an appropriate child seat (Owens, 2019). CRS use rates in taxi’s also provide an indication of CRS use rates in for-hire vehicles. Using crash data from New York City, Prince and colleagues found that CRS use was less than 6% in taxis involved in a crash in which a child was injured (Prince et al., 2019).

While challenges remain, CRS use in personal vehicles has largely been a public health success. Observational surveys indicate that in 2017, passenger vehicle CRS use was 97.9% among children < 1 year of age, 95.3% among children 1–4 years of age, 89.4% among children 4–7 years, and 84.4%.among children 8–12 years (Li & Pickrell, 2018). We expect use rates to be lower in ridesharing trips because of the widespread practice of exempting these vehicles in state laws, and because of the difficulties parents can face in using CRS in ride-sharing vehicles. Notably, ordering a rideshare that includes a company-provided CRS can substantially increase the cost of a trip. This may become an additional barrier to their adoption and use.

Pediatricians and injury prevention practitioners can reinforce the need for appropriate CRS use when using any vehicle, including those for hire, such as rideshare and taxis. Companies could demonstrate their commitment to safety by requiring CRS use regardless of the state law and providing a CRS when needed. As personal vehicle ownership declines and trips are increasingly likely to take place in ridesharing vehicles and taxis (Polzin et al., 2014), engineering solutions will be needed to provide CRS options that are easily carried and stored, or even built into vehicles. Such added convenience could encourage parents, caregivers and drivers to ensure child passenger safety in rideshare vehicles. In the longer term, a coordinated strategy is needed to address gaps in state legislation regarding CRS use in ridesharing vehicles.

This study is based on responses from the Harris Online panel, which is a non-probability sample. This means the results may be vulnerable to sampling biases such as self-selection. To adjust the sample to be nationally representative, a propensity weight was applied to each individual respondent’s data. However, it is possible that some bias remained in the sample, even after the weights were applied (Copas et al., 2020). This may be one reason why the prevalence of ridesharing in our study was marginally higher than what was reported in other surveys (Jiang, 2019).

CRS use reduces risk of serious injury or death for children in a crash (Arbogast et al., 2004). There is an urgent need for action to ensure that use of rideshare does not increase child injury risk. The first step is to determine accurate estimates of CRS use in rideshare vehicles, which is currently unknown, and identify barriers and facilitators of CRS use in such vehicles. Progress in personal mobility in the U.S. should not be accompanied by setbacks in child safety.

## Abbreviation

CRS: Child restraint systems
